# Mutation accumulation in *Tetrahymena*

**DOI:** 10.1186/1471-2148-10-354

**Published:** 2010-11-15

**Authors:** Patrícia H Brito, Elsa Guilherme, Helena Soares, Isabel Gordo

**Affiliations:** 1 Instituto Gulbenkian de Ciência, Oeiras 2781-901, Portugal; 2 Centro de Química e Bioquímica, Faculdade de Ciências, Universidade de Lisboa, Lisboa 1749-016, Portugal; 3 Escola Superior de Tecnologia da Saúde de Lisboa, Lisboa 1990 -- 096, Portugal

## Abstract

**Background:**

The rate and fitness effects of mutations are key in understanding the evolution of every species. Traditionally, these parameters are estimated in mutation accumulation experiments where replicate lines are propagated in conditions that allow mutations to randomly accumulate without the purging effect of natural selection. These experiments have been performed with many model organisms but we still lack empirical estimates of the rate and effects of mutation in the protists.

**Results:**

We performed a mutation accumulation (MA) experiment in *Tetrahymena thermophila*, a species that can reproduce sexually and asexually in nature, and measured both the mean decline and variance increase in fitness of 20 lines. The results obtained with *T. thermophila *were compared with *T. pyriformis *that is an obligate asexual species. We show that MA lines of *T. thermophila *go to extinction at a rate of 1.25 clonal extinctions per bottleneck. In contrast, populations of *T. pyriformis *show a much higher resistance to extinction. Variation in gene copy number is likely to be a key factor in explaining these results, and indeed we show that *T. pyriformis *has a higher mean copy number per cell than *T. thermophila*. From fitness measurements during the MA experiment, we infer a rate of mutation to copy number variation of 0.0333 per haploid MAC genome of *T. thermophila *and a mean effect against copy number variation of 0.16. A strong effect of population size in the rate of fitness decline was also found, consistent with the increased power of natural selection.

**Conclusions:**

The rate of clonal extinction measured for *T. thermophila *is characteristic of a mutational degradation and suggests that this species must undergo sexual reproduction to avoid the deleterious effects detected in the laboratory experiments. We also suggest that an increase in chromosomal copy number associated with the phenotypic assortment of amitotic divisions can provide an alternative mechanism to escape the deleterious effect of random chromosomal copy number variation in species like *T. pyriformis *that lack the resetting mechanism of sexual reproduction. Our results are relevant to the understanding of cell line longevity and senescence in ciliates.

## Background

Understanding the rate at which new mutations arise and their effects on fitness has been central in the development of many important theories in evolutionary genetics such as those that attempt to explain the evolution of sex and recombination [1], patterns of DNA sequence variation and molecular evolution in chromosomal regions with different recombination rates [2], the evolution of diploidy [3] and polyploidy [4], and the risk of small populations to become extinct [5]. Mutation accumulation (MA) is a classical experiment to estimate the rate at which deleterious mutations arise in populations and their effects on fitness [recently reviewed in [6]]. This approach is characterized by the propagation of replicates of highly inbreed and clonal populations over a long period of time in an environment where mutations over the whole genome are allowed to randomly accumulate without the purging effect of selection. Because different replicate lines will accumulate distinct mutations, the variance in fitness among lines is expected to increase over time. Fitness assays carried out with contemporaneous and ancestral populations may thus explain the phenotypic effects of the new accumulated mutations and can be used to provide estimates of the haploid genomic mutation rate per generation (U), the mean mutational effect (E(s)) [7,8], and the distribution of effects of new mutations (f(s)) [9]. Several model organisms, including some microbes, have been subject to such MA experiments and have permitted estimates of the rates and effects of mutations. MA experiments are expected to increase the genetic load and potentially lead to extinction due to fitness decline [10], as it has been observed, for example, in strains of *E. coli *[11] or in yeast [12].

Mutation accumulation studies have already been done with a wide variety of organisms, including viruses [13], bacteria [14,15], fungi [16], plants [17], and animals [e.g. [7,18]]. To our knowledge, however, experiments to estimate the rate and effects of mutations have never been performed with any of the diverse lineages of protists. Although Lynch and Gabriel [19] had previously estimated the mutation load of *Paramecium caudatum *this estimate was performed on data collected to determine the species maximum lifespan and therefore replicate lines were obtained from a diverse population and initial phenotypic variability was not set to zero as it is typical of MA studies.

Ciliates have a unique form of genomic information. As typical ciliate, *Tetrahymena thermophila *presents a marked nuclear dimorphism characterized by a diploid micronucleus (Mic) with five chromosomes that functions primarily as the germ line, and a somatic polyploid macronucleus (Mac) thought to be responsible for all gene expression during vegetative growth [20-23]. The Mac originates from the Mic and goes through several developmentally programmed DNA rearrangements such as site-specific fragmentation of the five chromosomes leading to 225 autonomous replicating pieces (ARP) that comprises a total of 104 Mb in length and more than 27000 predicted protein-coding genes [24-26]. Each of these ARP undergoes several rounds of replication until each locus is present at an average level of 45 copies per Mac genome [27,28].

**Figure 1:**
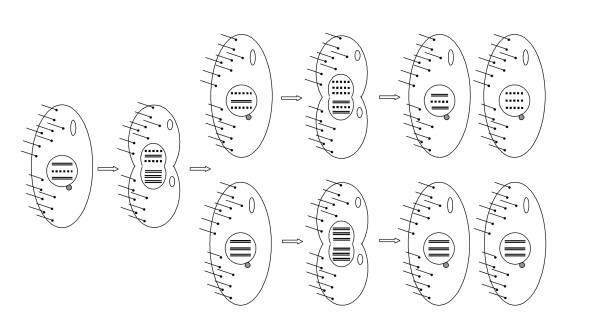
**Phenotypic assortment due to the amitotic divisions of the macronucleus**. Schematic representation of phenotypic assortment in the Mac genomes of *Tetrahymena *during asexual reproduction. The larger circle represents the macronucleus and small gray circle indicates the micronucleus, which is not shown in the dividing cells. For simplicity the Mac genetic content is represented by only three copies of one autonomous replicating piece (ARP) initially with two different alleles (straight double lines and dotted lines). As the process of phenotypic assortment occurs the frequency of heterozygous cells in the population gradually decreases until a completely homozygous population is form.

During vegetative growth the Mic divides following a regular mitosis while the Mac constricts and divides amitotically due to the absence of mitotic spindles and functional centromeres [23]. The major consequence of amitosis is the unequal partition of the genetic material between the two daughter cells, which causes a random segregation of alleles and an asymmetric division of the chromosomes (Figure 1). This unequal division of the genetic material after each cell division is expected to result in the tendency towards increased variance in macronuclear DNA content in the absence of a specific regulatory mechanism of copy number control [29-32]. It was suggested that this trend could be constrained by the elimination of the two extremes through suppression [30] or repetition [33] of complete rounds of macronuclear DNA synthesis between each cell division [34]. Although this cell cycle regulating mechanism has been observed it has not been formally demonstrated to be the mechanism that allows the cell to maintain its chromosomal copy number at the optimal level.

Another important consequence of the random segregation of the homologous chromosome copies at each cell division is the production of homozygous phenotypes from sexually-produced heterozygotes by the process of phenotypic assortment [35]. The general explanation for phenotypic assortment simply requires a randomly distribution of alleles between the two daughter cells [28,36]. The rate of assortment is dependent on the initial input ratio and on the number of assorting units and proceeds to homozygosity independently of the dominance relationships between alleles. The allele assortment rate has been estimated in *T. thermophila *for several loci and leads to a value of about 45 assorting units typical of the Mac of this species [27,28,36].

In this study we carry out a mutation accumulation experiment with the ciliate protozoan *Tetrahymena thermophila *with the aim to determine its somatic macronuclear (Mac) genomic mutation rate and the effect of new deleterious mutations on the mean population fitness. Even though experiments similar to this MA have been performed in the past [32,37,38], mainly to determine the possible immortality of ciliates [39], this is the first attempt to estimate the genomic mutation rate and effects of deleterious mutations in a protist. Due to the particular mode that characterizes the genetic segregation in the macronucleous of *Tetrahymena *that defies the rules of diploid Mendelian inheritance, we developed a simple simulation model to estimate both U - the rate at which copy number variation in the MAC occurs- and *s *- the mean effect on fitness caused by deviations of the optimum copy number. The MA experiment carried out in this study with *T. thermophila *was replicated with cell lines of *Tetrahymena pyriformis *with the aim to compare their rates of clonal extinction. The macronucleous of *T. pyriformis *is also fragmented into many ARP, each one highly amplified, and cell divisions are characterized by the amitotic segregation of alleles between the two daughter cells [31,40]. In contrast to *T. thermophila*, *T. pyriformis *organisms are amicronucleated, i.e. they lack the germinal micronucleus and are therefore unable to reproduce sexually with both germinal and somatic functions being carried out by the macronucleus alone. Most importantly, these organisms lack the ability to purge deleterious mutation through meiosis and conjugation, and therefore they provide an important contrast to the somatic MA occurring the in Mac of *T. thermophila*. Although during the course of the experiment *T. thermophila *accumulates mutations in both Mic and Mac nuclei, our phenotypic measures of fitness only detect the mutations accumulated in the macronucleus. Hence the results are comparable between species and they show that the mutation load in *T. thermophila *macronucleus is much greater than in *T. pyriformis*. *Tetrahymena *offers the opportunity to investigate (1) the establishment of the somatic vs. germ line during evolution process, (2) the advantage of having either sexual or asexual life cycles, and (3) the dynamics of and diploidy vs. polyploidy coexisting in the same cell/organism. Finally, *T. thermophila *is an important model organism in biomedical research and understanding the magnitude of its genomic mutation rate is critical to any evolutionary interpretation of its phenotypes.

## Results

### Mutation accumulation experiment and fitness assays

**Figure 2:**
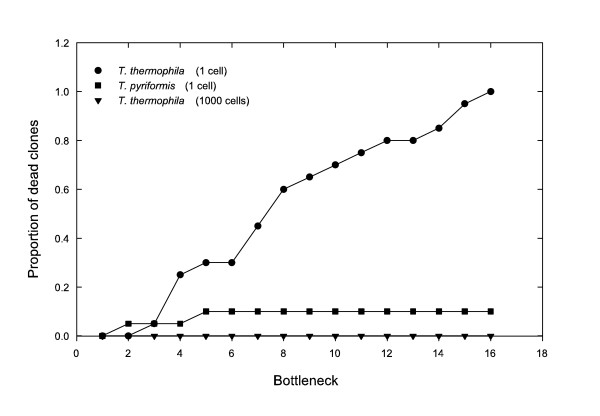
**Rate of clonal extinction of the mutation accumulation lines**. Data represents the cumulative total number of extinct clones as a function of the number of bottlenecks for *T. thermophila *sampled one cell every 48 hours (circles), and 1000 cells every 24 hours (triangles), and for *T. pyriformis *sampled 1 cell every 48 hours (squares).

The mutation accumulation (MA) experiment was carried out with 20 lines of *Tetrahymena thermophila*, all derived from the same clonal population that was derived from a single cell in the previous 24 hours, hence with no measureable phenotypic variation. Sampling of a single individual was performed every 48 hours, and was continued for 16 bottlenecks, after which all lines were extinct. During the course of the experiment these extinctions occurred at a rather constant rate of approximately 1.25 clonal extinctions per bottleneck (Figure 2) and the mean time to extinction of the 20 replicate lines was 8.55 ± 4.07 bottlenecks. The same experiment carried out with *T. pyriformis *gave strikingly different results. While the 20 MA lines of *T. thermophila *went extinct within a short period of 16 bottlenecks, *T. pyriformis*, on the other hand, showed a much higher robustness to extinction with only two clonal deaths during the same time period.

**Figure 3:**
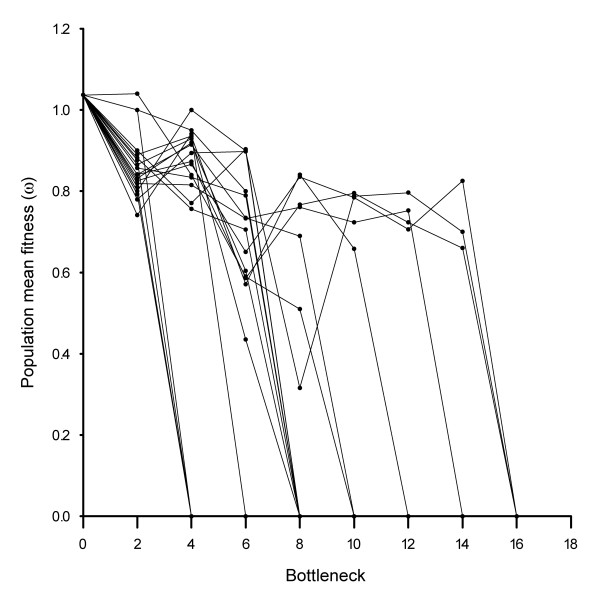
**Population mean fitness measured in the 20 mutation accumulation lines**. Each dot represents a fitness measurement and the lines connect successive measurements for the same replicate. For easiness of reading no error bars are represented. In a supplemental Figure (additional file 3: Fitness measurements for each population across bottlenecks) we show the fitness accompanied with their standard errors for each population separately.

**Figure 4:**
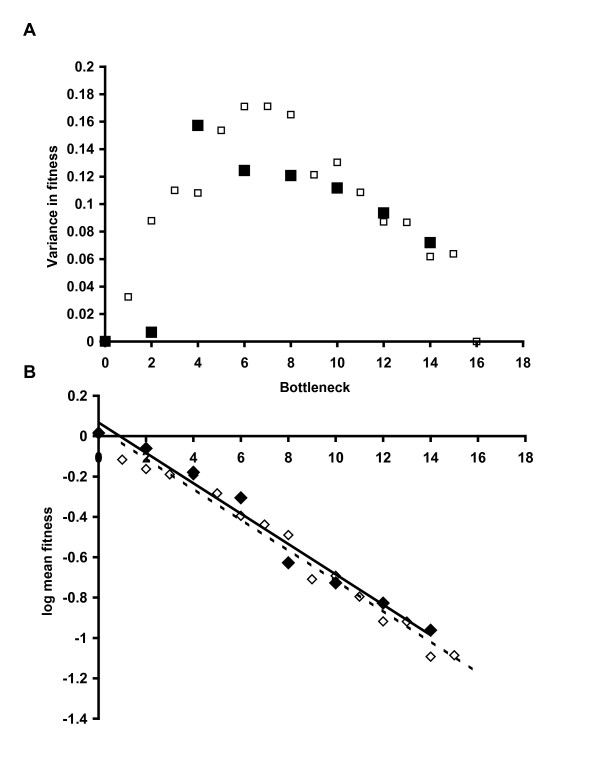
**Population mean fitness and variance for both empirical (solid marks) and simulated data (white marks) with bottleneck**. Our model simulates the experimental conditions of bottleneck size of one individual, and a mean copy number of 45 for each MAC locus. At each generation copy number varies with probability 1-e-U, and fitness of an individual with z copies was assumed to be W(z) = exp(-*s*|z-zm|), where *s *is the selection strength against changes in copy number (z-zm). Simulated data in this figure shows results for *s *= 0.16, U = 1.5. B shows the log transformation of the mean fitness with the number of bottlenecks. Fit for the rate of decrease was y = -0.0752x + 0.0676; r^2 ^= 0.976 for empirical data and y = -0.0761x + 0.0435, r^2 ^= 0.9763 for the simulated data. Standard errors of the rate of decline were 0.005 and 0.003 respectively, which leads these two regressions not significantly different.

**Figure 5:**
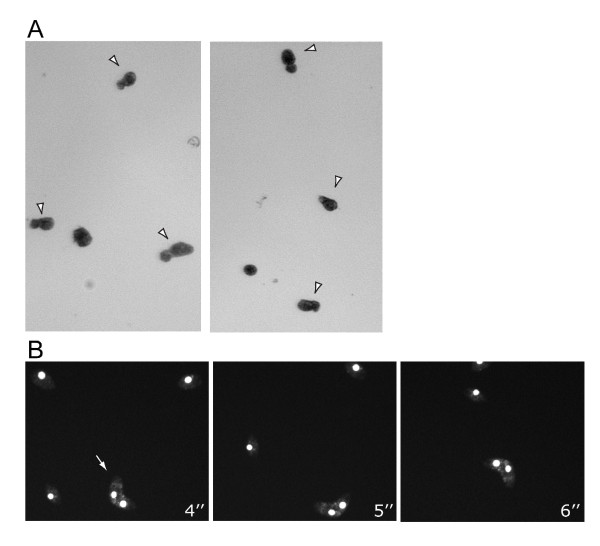
**Examples of mutant phenotypes of *Tetrahymena thermophila *cells obtained as a result of the MA experiment**. (A) Stereoscope images of drops containing abnormally asymmetrically dividing *T. thermophila *cells from bottleneck 16 (arrowheads); (B) alive cells with DNA stained with Hoechst 33342 and directly recorded under the fluorescent microscope. Images in B are selected frames from the video presented in supplementary material (Additional file 2: Video of an abnormal phenotype of *Tetrahymena thermophila *due to the MA experiment). The arrow points to a cell that is showing abnormal longitudinal dividing plan and presents an altered circular swimming behavior.

Measures of competitive fitness determined for each population along the bottlenecks, provide more complete information of the mutation accumulation process. Fitness measurements for the 20 MA lines show an overall tendency for mean population fitness to decrease during the experiment, as is typical of MA experiments (Figure 3 and Figure 4). Variance in fitness during the development of MA lines increases initially due to the likely accumulation of different mutations in each line. After bottleneck six, it stabilizes and then later decreases due to the increased number of extinct lines classified as having fitness of zero. At the end of the experiment (bottleneck 16), no variation in fitness between populations is obtained since all lines become extinct. Mean and variance of population fitness estimated excluding extinct lines are presented in a supplementary fig. S1 (Additional file 1: Population mean fitness and variance with bottlenecks). Most importantly, although the general tendency is for average fitness values to decrease within populations, occasional recoveries were detected in some lines. This is precisely what is expected to occur if a mechanism related to the variation in chromosomal copy number (which can either decrease of increase at each cell division, see below) is occurring. In fact similar dynamics can be observed in the simulation model that we developed. The model assumes that copy number variation is the key mechanism leading to the eventual extinction of all lines. More generally, throughout the experiment there was a decrease in fission rate, with an increased probability of the appearance of abnormally dividing cells with an altered plan of cell division or asymmetric divisions (Figure 5 and Additional file 2: Video of an abnormal phenotype of *Tetrahymena thermophila *due to the MA experiment). These are common phenotypic observation in experiments of this type and have been documented in the literature as evidence of macronuclear deterioration [32]. We also noticed that fitness measures taken prior to extinction were not predictive of the probability of extinction in the following bottleneck. We interpret this as further evidence for the effect of random drift in accelerating the process of macronuclear deterioration, a conclusion that contradicts what would be expected if viability were under the effect of selection. The efficacy of natural selection in preventing fitness decline depends on the population size. Estes et al [41] showed a strong effect of population size in the population mean fitness estimated from MA lines *C. elegans*. Lines that are kept at a slightly larger size had higher levels of mean fitness than lines kept at size one. By increasing the bottleneck size, the efficiency of selection against mutations of large effect will be improved. To evaluate the effect of bottleneck size in the rate of clonal extinction, we performed an additional experiment where 20 lines of *T. thermophila*, all derived from the same founding clonal population, were grown in the same conditions as the initial MA lines, except instead of transferring 1 single cell every 48 hours we performed daily transferences of 1000 cells. During the course of this experiment none of the replicate lines become extinct (Figure 2), and we did not detect any decay in the absolute fitness (data not shown). This strongly suggests that such an increase in the effective population size is enough for natural selection to act on the macronucleus rescuing populations of *T. thermophila *from extinction.

### Simulations

**Figure 6:**
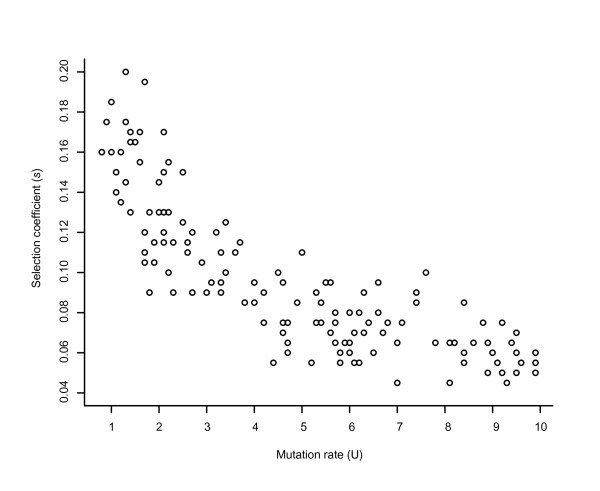
**Parameter space of U (mutation rate) and s (selection coefficient)**. Data presented represents the parameter space of U and s for which our model retrieves values of number of extinctions, and mean and variance in extinction time compatible with the empirical data (20 extinctions, mean extinction time of 8.55 bottlenecks, and variance of 16.15). Data on this figure correspond to simulated data for which 1 in 20 replicates obey to the following restrictions: 20 extinctions, mean extinction time between 6.5 and 10.5, and variance between 12 and 20. This set of simulations were carried out for U between 0.1 and 10 every 0.1, and s between 0.02 and 0.20 every 0.005.

Postulating that copy number variation is the key to explain the fast extinction observed, we developed a model to estimate the rate and mean effects of copy number variation in the population mean fitness. The model simulates as close as possible the experimental conditions of the MA experiment and assumes that the evolutionary consequences of a mechanism of variation in chromosomal copy number is enough to explain our experimental results. First the model and its parameters should be consistent with the rate of clonal death observed in *T. thermophila *(Figure 2) and second those estimated values of U and s should provide a pattern of mean fitness decline and fitness variance across bottlenecks similar to that observed in the experiment (Figure 4, solid marks). We performed an exhaustive search of the parameter space defined by U and s in order to characterize the set of parameters that generate data consistent with the empirical observations for clonal death: 20 extinctions in 20 populations and 16 bottlenecks, and a mean and variance in time to extinction of 8.55 and 16.15 bottlenecks, respectively (see Methods). Figure 6 describes the parameter space that corresponds to values of U and s for which the probability of obtaining data similar to the experiment (20 extinctions, mean time to extinction between 6.5 and 10.5, and variance between 12 and 20) is higher than 0.05. Our results show that both large values of U associated with small values of s, or smaller values of U associated with large values of s, can provide reasonable fits to the main features of the data. This shows that U covaries strongly with s which makes it difficult to estimate these parameters independently. In fact, assuming a slightly different model, Lynch obtained that the decline in viability over time depends on the product Us. We further explore this space for a set of parameters whose fitness dynamics was close to the one observed, and the parameter values of s = 0.16, U = 1.5 showed a good fit with the observed fitness dynamics, where both the rates of mean fitness decrease are not significantly different (Figure 4). We should note that these estimates of U and s should be taken as ball park estimates. The mutation rate of U = 1.5 corresponds to our best estimate of the somatic mutation rate for the whole macronucleus of *T. thermophila *and can be interpreted as the probability of somatic chromosomal lost/gain per Mac genome per generation. Considering that this species has on average 45 copies of each chromosomal replicating unit this estimate becomes 0.0333 per haploid Mac genome per generation. The same set of parameters in a model that simulates the experimental conditions of transferring a larger number of cells leads to the expected result of no clonal extinction similar to the empirical results (data not shown).

### Q-PCR assays

Our model also suggests that a difference in the level of Mac polyploidization could be sufficient to explain the rather distinct results obtained for both *T. thermophila *and *T. pyriformis*, where the later is expected to have a larger macronuclear copy number. To investigate the differences in copy number per cell between these two species we carried out Q-PCR for three single-copy genes per haploid genome (actin, alfa-tubulin, and elongation factor 1 alpha). In this analysis we assume similar amplification efficiencies between species and independent inferences of the ploidy level by each gene. The results indicate that *T. pyriformis *has on average 2.1 more copies per ng of DNA than *T. thermophila *(Table 1). Because the ratio of the number of cells of *T. pyriformis *to *T. thermophila *per ng of DNA after DNA extraction was 0.37 (i.e. each cell of *T. pyriformis *was estimated to have on average more 2.7 ng of DNA per cell) we estimated that *T. pyriformis *has on average more 5.6 copies per cell than *T. thermophila*. This result is larger but on the same order of magnitude with other rough estimates of macronuclear DNA content for several *Tetrahymena *species that suggest a mean ratio of the ploidy level between *T. pyriformis *and *T. thermophila *to be approximately 1.5 [42].

**Table 1:** Relative proportion of *T. pyriformis *gene copy number per ng of DNA relative to *T. thermophila*

	Actin	alpha-Tubulin	**Ef1a**^**1**^	
	2.084	1.607	1.797	

	2.739	2.021	2.081	

Mean	2.411	1.814	1.939	2.055

SE	0.327	0.207	0.142	0.182

^1^Elongation factor 1 alpha

## Discussion

The rate at which *T. thermophila *clones become extinct through continuous reduction in population size imposed by the strong bottlenecks is characteristic of a mutational meltdown [43]. During the course of the experiment, a synergistic interaction is created between population size and the random effect of genetic drift, and this promotes the build up of the population mutation load which, in its extreme, causes a mutation meltdown and drives populations to extinction [19]. In fact, although some extinctions have been previously observed in mutator strains of bacteria [11] and yeasts [12], no such high rate of extinction was observed in MA experiments with other organisms. In our experiments *T. thermophila *showed a decline rate of 1.25 clonal extinctions per bottleneck, a value that is at least two and three orders of magnitude faster than the rates of 0.01 and 0.06 obtained in the yeast and bacteria experiments. It is unlikely that the fast rate observed in *T. thermophila *is due solely to the accumulation of random deleterious mutations, such as nucleotide changes, transpositions and/or small insertions-deletions as observed in other organisms. More plausible is that this rate of extinction reflects the process of gain and loss of chromosomes that is characteristic of the amitotic division of the macronucleous of *T. thermophila*. Theory predicts that the accumulation of deleterious mutations in MA lines is accompanied by a steady decrease in the mean population fitness [10]. However, in our study we observed occasional recoveries of the mean population fitness (Figure 3 and Additional file 3: Fitness measurements for each population across bottlenecks), which can only be obtained when the rate of back mutations is sufficiently high for offspring to be fitter than the parental generation [10]. In our simulations we mimic these recoveries when we assume no bias for the probability of gain and loss of chromosomal copies in the amitotic division, a condition that still lacks empirical confirmation.

Lynch and Gabriel [19] analyzed the data from Takagi and Yoshida [32] and, by regressing the fraction of extinct or non-dividing cells on the number of cell divisions estimated the macronuclear mutation load (*μs*) of 0.0009 for a clone of *P. caudatum *- a ciliate that shares with *Tetrahymena *many of the genetic features associated with the presence of two nuclei and the amitotic division of the macronucleus. There are several differences both in the experimental design used by Takagi and Yoshida and the model used by Lynch and Gabriel to reanalyzed the data from that experiment. Takagi and Yoshida followed the evolution of 10 clones, replicated 3 times each, derived from a large population of *P caudatum*. Each of these lines were propagated with daily transfers of one cell, and occasionally replaced by members of sister sublines, a procedure we have not done and is not typical in MA experiments. Lynch and Gabriel applied a model to the data obtained by measuring the fraction of clonal populations with dividing cells during the experiment. Lynch and Gabriel model assumes that mutations occur following a Poisson process, just as we do in our model, and that each mutation causes a negative effect on fitness, s, that is irreversible. This was not assumed in our model. In our model each mutation causes a deviation from optimal copy number (W(Z) = exp(-s|Z-Zm|), see simulation model in Methods section), which causes a negative fitness effect that can be reverted. Under Lynch and Gabriel model, no selection occurs in the experiment and a linear decline in log mean fitness is expected over time, which translates into an estimate of Us, the mutational load. Lynch and Gabriel point out that since several simplifying assumptions are done in the model, including lack of back-mutation and lack of selection, this estimated is likely downwardly biased. Importantly not only Takagi and Yoshida did not perform a classical MA experiment but also they did not measure competitive fitness. Lynch and Gabriel performed a regression of the fraction of clones with dividing cells on the number of cell divisions to get a rough estimate of the mutational load in *P. caudatum *of 0.0009. If we applied the same model used by Lynch and Gabriel on our data of the extinction pattern (as used for *P. caudatum*) we obtain an equivalent value for the macronuclear mutation load in *T. thermophila *of 0.004. This value is four times larger than the one estimated for *P. caudatum*, which may reflect differences in macronuclear gene content between these two species and differences in experimental design. This load measures the proportional reduction in fitness due to deleterious mutations and lies within the range of *μs *(0.02 to 0.0002) estimated for eukaryotes [19]. Interestingly, a 0.002 rate of clonal death was independently estimated by Merriam and Bruns [27] for *T. thermophila*. These authors demonstrated that this rate is consistent with the rate of phenotypic assortment of a single gene, and suggested that the lower the copy number in the macronucleus, the higher the probability of expressing a deleterious allele in the homozygous form, and thus kill the cell. Our proposed mechanism also assumes that an increase in copy number in the macronuclear genome would provide higher robustness to clonal extinction. In fact, the selection coefficient (s) against a change in copy number away from an optimum value (Zm) may depend upon the absolute number of copies. It is easy to imagine that a cell carrying 44 copies of a gene whose optimum number of copies is 45 will have a lower fitness relative to a cell carrying 89 copies whose optimum value is 90. If this is the case then we can easily understand the difference in results of the MA of *T. thermophila *and *T. pyriformis*. For the same rate U a smaller s will lead to a much lower level of extinctions in the same time period (Figure 6). Lynch and Gabriel (1990) also speculate that the high longevities attributed to the amicronucleated *Tetrahymena *could be associated with an increase in mean extinction time when the effects of mutations are highly variable relative to their expectations - exactly what is expected when the gain and loss of chromosomal copies is achieved through a mechanism that is random relatively to the DNA composition of what is transferred.

The ecology associated with natural populations of *Tetrahymena *imply that they periodically suffer drastic fluctuations in population size [44] and hence suggests that *T. thermophila *species must recurrently undergo sexual reproduction in order to avoid the deleterious effects seen in our laboratory experiments due to extreme and periodic size reductions. The robustness of *T. pyriformis *to the accumulation of deleterious mutations in the MA lines, on the other hand, requires that species that lack the resetting mechanism of sex must take advantage of another mechanism that would confer protection from the deleterious effects of randomly accumulated mutations. The dependence of the rate of phenotypic assortment on the level of copy number suggests that longevity would benefit from (i) high copy number that allows for longer assortment times [27,39], and (ii) shorter chromosomes sizes so that the linkage between deleterious and beneficial mutations can be broken down thus promoting more efficient selection. Our computer simulations show that the observed rate of clonal death can be obtained simply as a consequence of the evolutionary dynamics of an unfaithful mechanism of copy number segregation that, in the face of strong genetic drift, is unable to maintain the cell line at an optimum number of copies.

One important result from our study is the suggestion that the mechanism that regulates copy number in the macronucleus is natural selection acting on the diversity generated by a stochastic process of gain and loss of chromosomes. It has always been a puzzle how the continuous amitotic mechanism of macronuclear division does not lead to massive chromosomal imbalances and death from aneuploidy For this reason, a regulatory mechanism of copy number control through replication was favored [45,46]. It was observed that a cell-cycle mechanism such as the one proposed by Preer and Preer [46] can rescue the cells from extreme chromosomal imbalances. But the fact that once started replication would duplicate all DNA content present in the macronucleus [33] strongly suggests this mechanism would be a last resort solution.

This is the not the first time that a stochastic model is advocated to explain the chromosomal segregation in ciliate macronucleus [47]. However, unlike previous models we postulate that changes in chromosome copy number, even the smaller changes compatible to what is expected from each cell fission [42], carry fitness costs that are seen by selection. We can therefore hypothesize that under large effective population sizes the probability of population extinction through genetic imbalances should be small. In fact, once we allow for selection to be effective (by increasing Ne) no such rate of extinction is observed, suggesting that the rate of extinction was due to ineffective selection in the face of intense genetic drift. Our model does not explain assortment of rDNA given these genes are present in such high copy numbers unless these copies occur in synteny. Interestingly, Merrian and Burns [27] estimated a rate of assortment for rdnA2 [a mutation in the gene for ribosomal DNA that is present in 10000 copies, [48]] that is similar to the assortment rate of other genes maintained at 45 copies. It was suggested an epigenetic regulation independent of the assortment of other genes could explain the regulation of these highly amplified gene copies [45,49].

## Conclusion

The aim of this study was to estimate the somatic mutation rate of *T. thermophila *by performing a classic experiment of mutation accumulation. We observed a rate of clonal death and a fitness dynamics that are consistent with a mechanism of gain/loss of chromosomes, as is characteristic of the amitotic division of *Tetrahymena *macronucleus. The high similarity of estimates available for the mutation load in *T. thermophila *also strengthens the hypothesis that the mechanism responsible for the accumulation of deleterious effects is dependent on a species-specific feature such as chromosomal copy number. We suggest that copy number and the rate of chromosomal gain/loss at each amitotic division are crucial to understand macronuclear degeneration, cell line longevity, and senescence in ciliates. It also renders the hypothesis of longevity controlled by programmed cell death unlikely.

## Methods

### Strains

*Tetrahymena thermophila*, B2086, mating type II, and *Tetrahymena pyriformis *GL-C; these strains were obtained from the *Tetrahymena *Stock Center at Cornell University http://tetrahymena.vet.cornell.edu.

### Mutation accumulation experiment

At time T0 twenty lines were established from single cells all originated from the same founding clonal culture. Because we were interested in estimating the somatic mutation rate, our initial cell was randomly selected from a culture of mature cells that were grown at large population sizes in standard conditions in the lab. This allowed us to start the experiment with clonal populations that had already gone through phenotypic assortment and experienced the effect of natural selection. The twenty cells were incubated and let to grow in individual drops of 30 μl of rich medium (SPP: 1% proteose peptone, 0.1% yeast extract, 0.22% glucose, and 0.003% EDTA ferric sodium salt) for 48 hours that corresponds to approximately 16 generations in the ancestral cell population. Drops were maintained on sterile Petri dishes placed in humid boxes at 30° and 28° for *T. thermophila *and *T. pyriformis*, respectively, which are the temperatures normally used in laboratory to grow cultures of each species. These lines were periodically bottlenecked (every 48 hours) by randomly sampling one individual cell and transferred it to a fresh medium drop. This procedure was repeated for 20 bottlenecks or until extinction of all lines. Every two bottlenecks populations were frozen and fitness was measured through competition with the ancestral type. Whenever a drop was detected with no more remaining live cells, a new cell was taken from the previous drop in an attempted to maintain the culture line, in most cases these attempts were not successful and a the culture line would be considered extinct.

To evaluate the effect of bottleneck size in the extinction rate obtained for *T. thermophila *an additional experiment was performed where twenty lines of *T. thermophila *all derived from the same founding clonal population, were grown in the same experimental conditions as above but with daily transferences of 1 μl that corresponded to approximately 1000 individual cells.

### Fitness assays

Mean and variance in population fitness were measured in the ancestral population and in the MA population replicates every two bottlenecks until extinction of all lines. Fitness was measured through competition of the experimental samples of *T. thermophila *with a *T. thermophila *CU522 strain expressing GFP under the promoter MTT1 (metallothionein 1 protein) dependent of cadmium chloride (Seixas *et al *unpublished data) and relative fitness of the evolved strains relative to the ancestral ones was interpolated from the competition assays done with the GFP-expressing cultures. Competitions were performed by putting one cell of each strain in a single medium drop and letting it incubate at 30°C for 48 hours. Initial cell number of each genotype (Ni) was 1, and final densities (Nf) were estimated from three sets of 10 μl fluorescent induced cultures collected from each competition drop and mounted in slides that were observed with a Leica DMRA2 microscope. GFP expression was induced by 2 μl of CdCl_2 _(0, 5 μg/ml) to each drop for one hour, and slides were prepared by adding 10 μl of extraction buffer and 14 μl of 2% paraformaldehyde to the 10 μl culture sample, that were let to dry for 30 min at 30°C, and later mounted in a slide with DABCO. All competitions were performed in triplicate. Live images from *Tetrahymena *cells were obtained with Leica DMRA2 microscope, and nuclei were stained with Hoechst 33342 for 15 minutes at 30°, at a final concentration of 2 μg/μl.

### DNA extractions and Q-PCR assays

Total DNA was extracted following protocol of Gaertig *et al *[50] that applies a standard phenol-chloroform and ethanol DNA extraction method with a final overnight RNA digestion with RNase A. Q-PCR (quantitative real-time PCR) was used in this study to quantify the relative differences in Mac copy number between *T. thermophila *and *T. pyriformis*. It is expected that differences in amplification of single copy genes will reflect differences in copy number whenever the two initial DNA concentrations are kept equal and the genes are constitutively expressed. The genes selected (Actin, alpha-tubulin, and Elongation factor 1 alpha) are all single copy genes in the haploid genome [51-53] and if located in different Mac chromosomes, each one would be an independent estimation of the level of duplication. Primers were designed with the help of Primer Express 3.0 (Applied Biosystems, Foster City, CA) in highly conserved regions between the two species so the same primer pairs could be used in the PCR amplifications and similar amplification efficiencies could be assumed. The primer sequences and the DNA sequences of the above genes, obtained from Genbank for both species, are listed on Table 2. Real-time PCR was performed with Fast SYBR Green Master mix (Applied Biosystems, Foster City, CA) chemistry in an ABI 7900HT Fast Real-Time PCR system. Initial PCR amplifications were carried out to optimize the reactions and insure that single products were amplified in each reaction. Initial template concentrations for both species were quantified with NanoDrop 1000 Spectrophotometer (Thermo Scientific) and the appropriate dilutions were done so equal concentrations were used in the Q-PCR reactions. Serial dilutions of the initial DNA concentration (25X, 5X, 1X, 0.2X, 0.04X) were performed on both species DNA templates that were used to build calibration curves of DNA concentration versus amplification efficiency. Q-PCR reactions were carried out in 384-well plates (Applied Biosystems) and individual reactions of 10 μl contain 5.0 μl of Fast SYBR Green Master mix (1X), 2.0 μl of each primer (0.25 μM) and 1 μl of DNA template at various concentrations. The default thermocycler program was used.

**Table 2:** Sequence Genbank accession numbers and primers used to assay gene copy number in *T. thermophila *and *T. pyriformis*

	Primers	GenBank Acession number
Actin	F: TCCCATTGAACACGGTATTGTC	*T.thermophila*: AY315823
		
	R: TGTAGAAGCAATGATGCCAGATCT	*T.pyriformis*: X05195

alpha-	F: ACGCCAAGAGAGCCTTCGT	*T.thermophila*: M86723
		
Tubulin	R: CGGAGAATTCACCTTCTTCCAT	*T.pyriformis*: X12767

Ef1a^1^	F: CGGTTTCAACATTAAGGGTGTCT	*T.thermophila*: CH670356
		
	R: GGCATCGGAAGCGACATT	*T.pyriformis*: D11083

Q-PCR assay was done twice for each gene and in each experiment each sample was run in quadruplicate. The threshold cycle (C_T_) for each well was calculated following the instrument instructions. Standard curves were prepared for DNA templates of both species and the relative differences in copy number (level of polyploidization) was estimated for each gene from the ratio of the averaged C_T _obtained for *T. pyriformis *and *T. thermophila *at equal DNA concentrations (1X).

### Simulation model

We built a model that simulates the experimental conditions of the mutation accumulation experiment as well as the variation in the chromosome copy number that occurs at each cell division due to the amitotical division of the Mac genome. Cells in our model are characterized by their mean chromosomal copy number (Z), and their fitness (viability) is solely dependent on how Z deviates from an optimal value (Zm). A population starting from a single cell would have Z=Zm = 45 copies in the macronucleus genome, and for simplicity this initial cell does not carry any recessive deleterious mutations. At every generation the number of copies either increases or decreases, with equal probability, at a rate U according to a Poisson distribution. Note that U parameterizes variation in copy number and not rate of acquisition of point mutations, as typically assumed in other organisms where mutation accumulation experiments have been performed. The relative fitness of an individual is determined by W(Z) = exp(-s|Z-Zm|), where s is the selection strength against changes in the copy number, and Z-Zm is the distance between that individuals' chromosomal copy number and the species' optimal copy number. This fitness landscape is the simplest function reflecting stabilizing selection for optimal copy number, and s is the effect on fitness of a change in one copy from the optimum number. When s is small this is the same as the effect of a single deleterious point mutation in classical models of MA [6]. Every generation population size fluctuates with reproduction (by duplication), mutation and viability selection. We let the population evolve until stationarity followed by a bottleneck of a randomly selected individual, or until extinction. The simulation ends after 16 bottlenecks or until all replicate lines are extinct. We explored our model to determine the parameter space of U (mutation rate towards increase or decrease in chromosomal copy number) and s (selection strength against changes in copy number) that recovers results compatible with the empirical observations, namely the number of extinctions observed in the 20 populations over the 16 bottlenecks, the mean and variance in extinction time observed experimentally. Specifically we initially searched a large parameter space defined by U in the interval [0.1; 12.0] and s in the interval [0.01; 1.0], in spaces of 0.05 for each of the parameters. This allowed us to eliminate combinations of U and s that lead to simulation results where the pattern of extinction in the experiment was not produced. From this search we restricted our parameter space to new intervals for U and s that were now searched in smaller spaces, for compatibility with the extinction pattern of the data. This involved and extensive search for U between 0.1 and 10.0, every 0.1, and s between 0.02 and 0.20, every 0.005. For the same parameter values of U and s, the simulation run was replicated 20 times. With this approach we can delimit the range of values of U and s that in our model produce results for number of extinctions, mean and variance of extinction time, that are compatible with the empirical results. The dynamics of fitness decline obtained for each set (U, s) compatible with the empirical results for the rate of extinction can then be determined in the simulations and compared with the fitness dynamics measured experimentally.

## Authors' contributions

IG and HS conceived the project, PHB, EG performed the experiments, and PHB, IG and HS analyzed and wrote the paper. All authors read and approved the final manuscript.

## Supplementary Material

Additional file 1 **Population mean fitness and variance with bottlenecks**. Variation in population mean fitness and variance along the experiment excluding the extinct replicate lines. Click here for file

Additional file 2 **Video of an abnormal phenotype of *Tetrahymena thermophila *due to the MA experiment**. Live images from *T. thermophila *cells with DNA stained with Hoechst 33342 and directly recorded with a Leica DMRA2 microscope. This video shows an example of a cell with an abnormal longitudinal dividing plan and an altered circular swimming behavior. Click here for file

Additional file 3 **Fitness measurements for each population across bottlenecks**. Each graph represents estimates of the population mean fitness (ω) measured every two bottlenecks for each replicate line. The error bars represent 2*SE (standard errors). Click here for file
