# Supplementary figures and images for: Mutation accumulation in Tetrahymena
Source: BMC Evol Biol. 2010 Nov 15;10:354. doi: 10.1186/1471-2148-10-354 (PMC2998532; doi:10.1186/1471-2148-10-354)

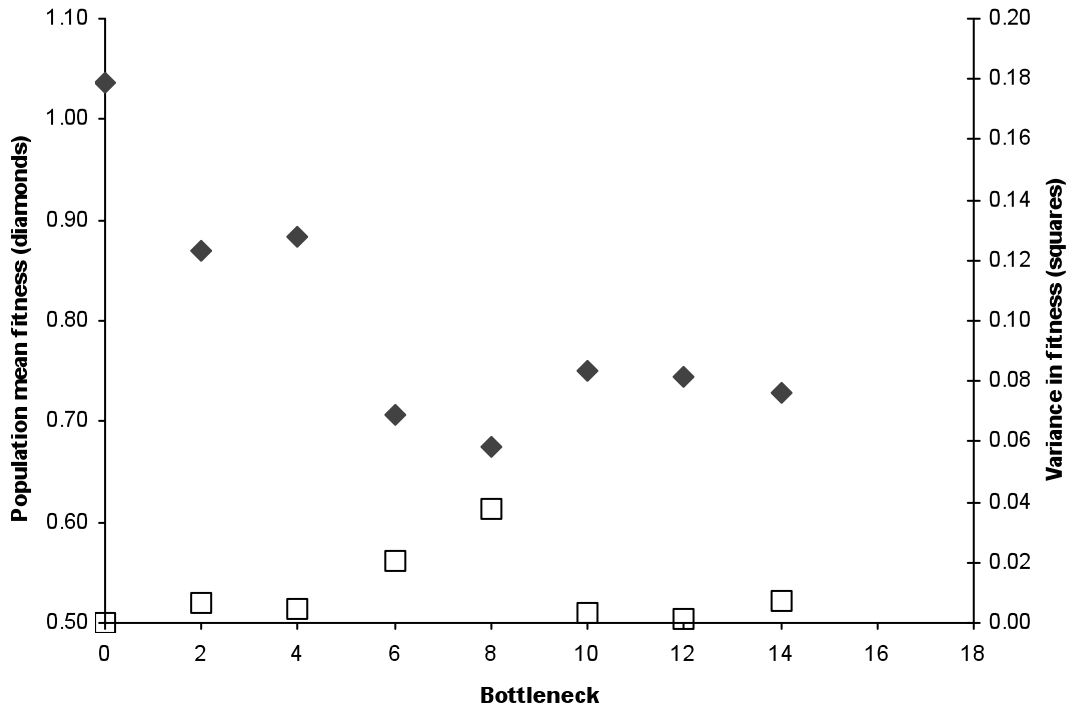

Supplement: Additional file 1 — Population mean fitness and variance with bottlenecks. Variation in population mean fitness and variance along the experiment excluding the extinct replicate lines. [file 1471-2148-10-354-S1.PDF]
