# Supplementary material for: Mutation accumulation in Tetrahymena
Source: BMC Evol Biol. 2010 Nov 15;10:354. doi: 10.1186/1471-2148-10-354 (PMC2998532; doi:10.1186/1471-2148-10-354)
Supplement: Additional file 3 — Fitness measurements for each population across bottlenecks. Each graph represents estimates of the population mean fitness (ω) measured every two bottlenecks for each replicate line. The error bars represent 2*SE (standard errors). [file 1471-2148-10-354-S3.PDF]

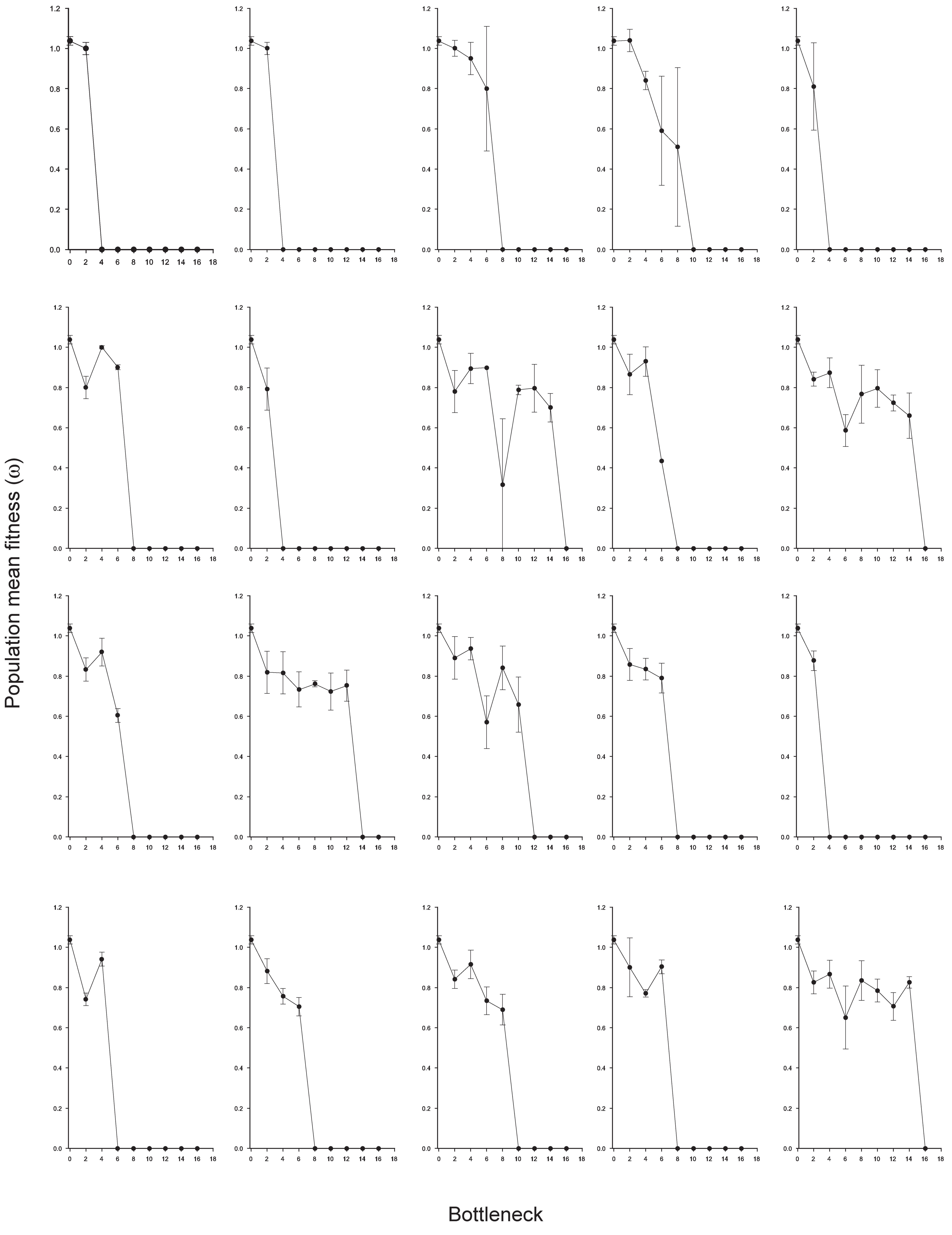
Population mean fitness (ω)
Bottleneck
0.0 0.2 0.4 0.6 0.8 1.0 1.2
0 2 4 6 8 10 12 14 16 18
